# Rif1-Dependent Control of Replication Timing

**DOI:** 10.3390/genes13030550

**Published:** 2022-03-20

**Authors:** Logan Richards, Souradip Das, Jared T. Nordman

**Affiliations:** Department of Biological Sciences, Vanderbilt University, Nashville, TN 37203, USA; logan.r.richards@vanderbilt.edu (L.R.); souradip.das@vanderbilt.edu (S.D.)

**Keywords:** DNA replication, genome stability, replication timing, Rif1, helicase, nuclear organization

## Abstract

Successful duplication of the genome requires the accurate replication of billions of base pairs of DNA within a relatively short time frame. Failure to accurately replicate the genome results in genomic instability and a host of diseases. To faithfully and rapidly replicate the genome, DNA replication must be tightly regulated and coordinated with many other nuclear processes. These regulations, however, must also be flexible as replication kinetics can change through development and differentiation. Exactly how DNA replication is regulated and how this regulation changes through development is an active field of research. One aspect of genome duplication where much remains to be discovered is replication timing (RT), which dictates when each segment of the genome is replicated during S phase. All organisms display some level of RT, yet the precise mechanisms that govern RT remain are not fully understood. The study of Rif1, a protein that actively regulates RT from yeast to humans, provides a key to unlock the underlying molecular mechanisms controlling RT. The paradigm for Rif1 function is to delay helicase activation within certain regions of the genome, causing these regions to replicate late in S phase. Many questions, however, remain about the intricacies of Rif1 function. Here, we review the current models for the activity of Rif1 with the goal of trying to understand how Rif1 functions to establish the RT program.

## 1. Introduction

Successful DNA replication depends on the accurate duplication of billions of bases every cell division cycle. DNA replication is largely regulated at the initiation phase, which includes the loading and subsequent activation of replicative helicases. First, the origin recognition complex (ORC) binds to cis-acting sites throughout the genome where DNA replication will initiate, known as replication origins [1]. ORC, together with Cdc6 and Cdt1, facilitates the loading of MCM2-7 double hexamers at replication origins in a process known as ‘helicase loading’ [1,2]. MCM2-7 is the replicative helicase but is loaded in an inactive state in late M and G1 phases of the cell cycle, thus marking all potential initiation sites [3]. In S phase, loaded MCMs must be activated through phosphorylation of the N-terminal regions of MCM2,4 and 6 by Dfb4 dependent kinase (DDK) [4,5]. Once activated, S phase cyclin dependent kinase (S-CDK) is required for the recruitment of additional initiation factors and assembly of the replisome [6,7,8,9,10].

Modeling and physiological studies indicate that helicase activation is the critical regulated step in DNA replication to establish replication kinetics and replication timing (RT—the time in S phase when a given DNA sequence is replicated) [11,12,13]. Helicase activation does not occur uniformly throughout the genome at the onset of S phase. Instead, helicase activation occurs continuously throughout S phase. Not all loaded helicases are activated during S phase. Even at a robust origin, efficiency of activation is 10% or less [14,15]. When measuring RT across multiple organisms and cell types, specific patterns emerge. For example, open and active regions of chromatin often replicate early in S phase, whereas condensed chromatin that is transcriptionally less active tends to replicate late in S phase [16]. On the surface, RT appears to bring order to chaos. RT allows thousands of independent initiation events to be orchestrated throughout S phase to ensure that the entire genome will be duplicated. The reality, however, is somewhat more random. 

While the population-level studies have established DNA replication timing is regulated at the level of genomic domains containing clusters of replication origins, a single-molecule study in human cells have revealed that initiation does not happen in a concerted manner utilizing distinct domains of frequently firing origins [17]. Instead, initiation occurs stochastically within larger zones that are arbitrarily allocated. For example, even the top 5% of initiation zones are only used in 11% of the population. This suggests that RT is both heterogenous and probabilistic. This model of RT is consistent with an in-silico model of replication kinetics assuming stochastic origin firing [18].

Replication timing has important implications in governing replication kinetics. While helicase activation appears to be the critical rate-limiting step for RT [12,13], the molecular underpinnings of helicase activation throughout the genome and the duration of S phase are still not fully understood. A mechanistic understanding of the factors controlling RT will be critical to the understanding of replication timing kinetics and genome stability.

## 2. The Biological Function of Replication Timing

**Figure 1:**
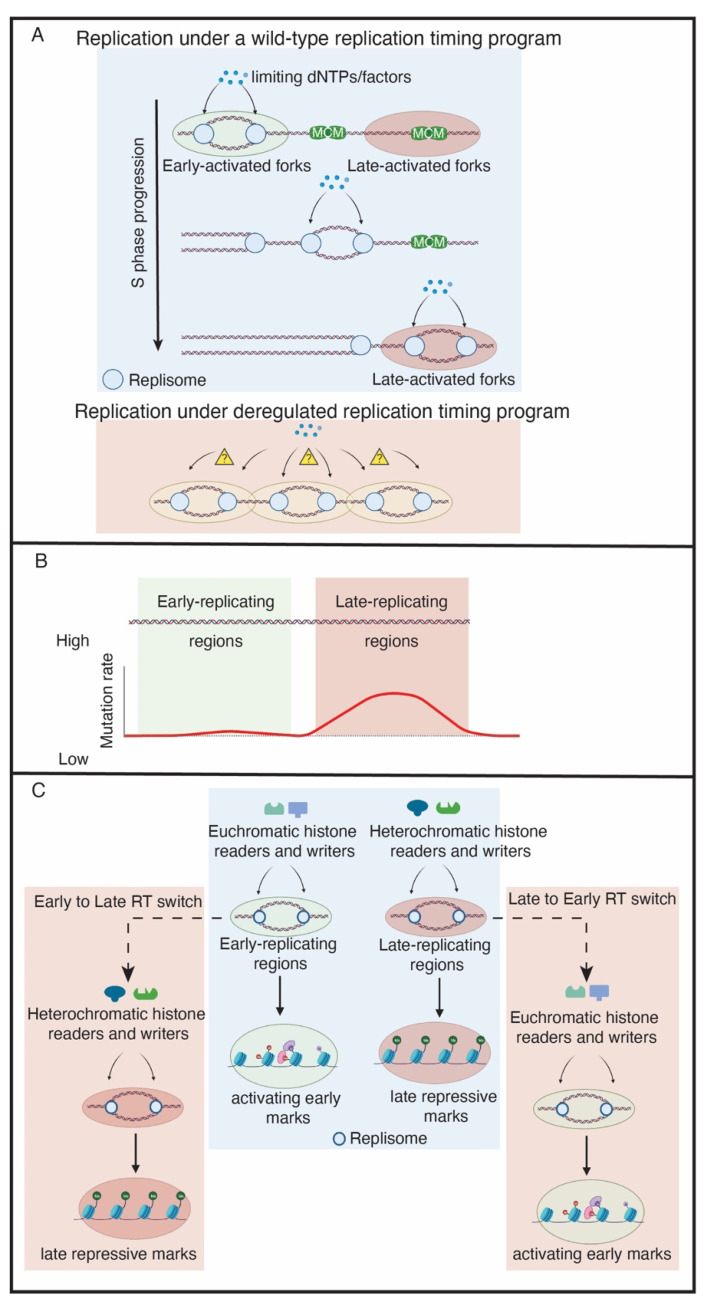
The functions of replication timing. (**A**) The replication timing program maintains a supply–demand equilibrium of limiting replication factors, which could be histones, replisome proteins or dNTPs. In a de-regulated replication timing program, an excess of origins may become activated, resulting in the pool of limiting replication factors to be depleted and DNA damage to occur. (**B**) Late-replicating regions have a higher mutation rate than early-replicating regions. (**C**) The replication timing program maintains the epigenetic landscape. In the wild-type example (blue box), replication timing preserves the epigenetic landscape. This is achieved by allowing euchromatic and heterochromatic histone readers and writers to be properly recruited to replication forks within early and late-replicating regions, respectively, during S phase. The pink boxes indicate de-regulated replication timing. In this example, a region switches from early to late replicating (E to L Switch) or late to early replicating (L to E switch). Consequently, the wrong histone readers and writers are targeted to those regions. This results in the epigenetic landscape changing upon replication timing disruption.

While genome-wide techniques such as Repli-seq, Timing Inferred from Genome Replication (TIGER), and optical replication mapping (ORM) have allowed us to measure RT with high precision and resolution across multiple organisms and cell types [17,19,20], the biological function of RT has been more difficult to ascertain. Several key studies, however, suggest that RT has at least two key functions: maintaining genome stability by ensuring the distribution of limiting factors across replication forks during S phase [12,13] and maintaining epigenetic information during replication [21,22] (Figure 1A).

In eukaryotes, the temporal order of origin firing defines the RT program. The low levels of the two CDK targets Sld3 and Sld2, their binding partner Dpb11 and the DDK subunit Dbf4 are limiting for replication initiation in budding yeast and Xenopus [12,13]. Overexpression of these four factors causes early firing of late origins and increases the speed of S phase [12]. Abolishment of the RT program in this case leads to a severe growth defect and activation of the checkpoint response. This is likely due to the exhaustion of limiting replication factors such as dNTPs and establishes the importance of RT to conserve limiting pools of replication factors to prevent genome instability [12]. Therefore, the RT program allows limiting factors such as dNTPs and histones to be distributed throughout S phase and avoids the exhaustion of these factors, which would trigger DNA damage and genome instability.

In addition to the genome instability caused by disruption of the RT program, the rate of mutation occurring in a genomic region is correlated with its RT in yeast, Drosophila, rodents and humans, with earlier replicating regions having a lower mutation rate than their late-replicating counterparts [23] (Figure 1B). The higher frequency of Single Nucleotide Variants (SNVs) in late replicating regions also plays a major role in building up acquired drug-resistance in lung cancer cells [24]. In an evolutionary assessment of human–chimpanzee substitutions and human SNP density, the mutation rate, as reflected in recent evolutionary divergence and human nucleotide diversity, is found to be markedly increased in later-replicating regions of the human genome [25]. A better understanding of the molecular underpinnings of the RT program will have substantial implications in genome stability, disease, drug-resistance and evolutionary biology.

RT could also be a key factor in maintaining epigenetic state (Figure 1C). Early work in rat cells suggested a mechanistic connection between replication timing and chromatin packaging [21]. Exogenous plasmids microinjected in nuclei during early S-phase were significantly enriched for acetylated histones, while deacetylated histones were associated with the plasmids injected in late S-phase. More recently, it was shown in human cells that, if RT is perturbed, the distribution of heterochromatic histone modifications is altered in human cells [22]. Interestingly, there are two classes of heterochromatic regions that are either affected or unaffected by perturbing the RT program. The affected domains tend to be enriched for smaller H3K9me3 peaks. In contrast, unaffected domains form much larger and broad H3K9me3 domains [22], which may somehow protect these domains from alterations in RT. Additionally, the levels of active histone modifications, specifically H3K27ac and H3K4me3, are significantly depleted if RT is de-regulated [22]. Consistent with RT as a critical regulator of epigenetic maintenance, these changes in histone modifications coincide with S phase [22]. This suggests that DNA replication is important for epigenetic modifications and that RT could play a vital role in the propagation of epigenetic information. These findings, however, were limited to human cell lines [22]. It will be interesting to understand if these observations are subject to developmental regulation or if they hold true in other species. Directly coupling RT to epigenetic status represents a new and exciting function of RT, but the mechanism as to how this occurs requires further investigation.

## 3. Rif1 Is a PP1 Specificity Factor That Regulates RT across Species

Because RT is closely associated with chromatin accessibly [16,26,27] and RT shows considerable cell-to-cell variability [28,29], it would be easy to assume that RT is a passive reflection of chromatin accessibility. The biochemical regulation of RT is beginning to be mechanistically understood through the discovery trans-acting factors that actively regulate RT. This indicates that, while helicase loading and activation are influenced by chromatin accessibility, the execution of the RT program is an actively controlled process. One factor that regulates RT is Rap1 interacting factor 1 (Rif1). Rif1 was initially discovered in a yeast 2-hybrid assay for proteins that interacted with Rap1, an essential regulatory protein in budding yeast [30]. The first evidence that Rif1 could regulate RT, however, arose from a study in budding yeast where *rif1Δ* cells caused genomic regions proximal to telomeres, which are normally late-replicating, to replicate earlier in S-phase [31]. Later studies revealed that loss of Rif1 activity cause global changes in RT in fission yeast, fruit flies, mice and humans [32,33,34,35,36]. Therefore, determining the mechanism of Rif1’s activity is critical to understanding how RT is regulated.

While Rif1 is a key regulator of RT, it has additional functions in chromatin biology that are independent of its ability to control RT. In budding yeast, Rif1 interacts with Rap1 to control telomere length [30]. Rif1’s involvement in functional telomere maintenance, however, appears specific to budding yeast [37,38,39,40,41,42]. In mammalian cells, Rif1 is involved in DNA double strand break (DSB) repair. In this context, Rif1 is recruited to DSBs by 53BP1 which, together with other factors, prevents end resection. This ultimately inhibits homologous recombination and promotes non-homologous end joining (NHEJ) [43,44,45,46]. Exciting recent work has identified several key players and protein complexes that work with Rif1 at DSBs [47,48]. Interestingly, Rif1 has a 53BP1-independent function in protecting cells from ultrafine anaphase bridges that form as a result of unresolved centromeric catenanes [49]. Rif1 can also be recruited to stalled replication forks in a 53BP1-independent manner [50]. In this review, however, we will focus our attention RT-specific functions of Rif1.

The mechanism of Rif1 function in RT control was initially suggested through a genetic interaction with Cdc7 (*hsk1*) in *Schizosaccharomyces pombe* (*S. pombe*) [32]. Cdc7 is the catalytic subunit of DDK [51]. *hsk1* null cells are inviable due to the inability to activate the replicative helicase [52]. A screen for bypass suppressors of *hsk1* null cells revealed that deletion of *rif1* could restore growth to *hsk1* null cells [32]. This study also revealed that the origin firing throughout the genome was altered in *rif1* null cells. While this work made clear that Rif1 was a negative regulator of replication, the biochemical mechanism was still unclear. 

Although Rif1 regulates replication from yeast to humans, its sequence has diverged considerably [53]. One common feature of all Rif1 orthologs, however, is the presence of a PP1 binding motif [54,55,56,57]. The proposed biochemical mechanism for Rif1-dependent control of DNA replication is based on Rif1’s ability to bind Protein Phosphatase 1 (PP1) and direct PP1 activity towards specific substrates [54,55,57,58,59]. In the context of helicase activation, Rif1/PP1 targets MCMs to oppose DDK-mediated activation the helicase, thus preventing or delaying initiation of replication at specific start sites. There is a wealth of genetic data connecting Rif1 to helicase activation. For example, reducing DDK activity leads to a decrease in MCM4 phosphorylation and a loss in viability, both of which can be suppressed by deleting *Rif1* [55,58,59]. Furthermore, MCM4 is hyper-phosphorylated in yeast and Xenopus if Rif1 is absent or depleted [55,58,59]. It is surprising that, while Rif1 clearly regulates MCM phosphorylation levels, there is little evidence of a direct biochemical interaction. While several studies have used an IP-mass spec approach to identify Rif1-associated proteins, the MCM complex has not been identified [54,59]. This could be for several reasons. For example, the association of Rif1/PP1 with MCMs could be too transient to identify by IP (although covalent cross linkers were used in these experiments). Thus, how Rif1 is targeted towards, and associates with, loaded helicases is still an outstanding question.

While loss of Rif1 function suppresses a temperature sensitive (ts) allele of *Cdc7* [58], loss of Rif1 activity also suppresses ts alleles of *Dpb11*, *Sld3* and *Cdc45* alleles [56]. This observation suggests that Rif1 more broadly regulates helicase activation, perhaps beyond just controlling MCM phosphorylation levels. Both Sld3 and Cdc45 are ‘readers’ of MCM phosphorylation. Their recruitment to MCM hexamers is dependent on MCM phosphorylation [60]. Perhaps the increased MCM phosphorylation in the absence of Rif1 increases the efficiency of Sld3 and Cdc45 recruitment, increasing the probability of helicase activation even with limiting amounts of Sld3 and Cdc45. Loss of Rif1 activity also increases the phosphorylation level of Sld3 in G1 phase, possibly directly impacting the activity of Sld3 [56]. The suppression of *Dpb11* ts phenotype, however, is not as obvious. Dpb11 and Sld3 physically associate in a phospho-specific manner [12,61]. This interaction, however, is dependent on CDK rather than DDK. Perhaps the increase in MCM phosphorylation that occurs upon loss of Rif1 function results in a more efficient helicase activation step. In this case, increased helicase activation could drive Dbp11-dependent replisome assembly. 

In support of this model, single molecule experiments revealed that DDK phosphorylation of MCMs recruits multiple GINS and Cdc45 subunits [62] Furthermore, DDK is required for the efficient formation of a key intermediate complex of the replicative helicase and eliminating a subset of phosphorylation sites on MCM2-7 reduces the efficiency of replicative helicase formation. This model has interesting implications on RT, where Rif1 also contributes to the balance of MCM phosphorylation during helicase activation. The phosphorylation of the N-terminal tails of MCMs correlates with the efficiency of helicase activation; therefore, this could provide a biochemical mechanism for Rif1-mediated delay of helicase activation and ultimate control of RT [62].

**Figure 2:**
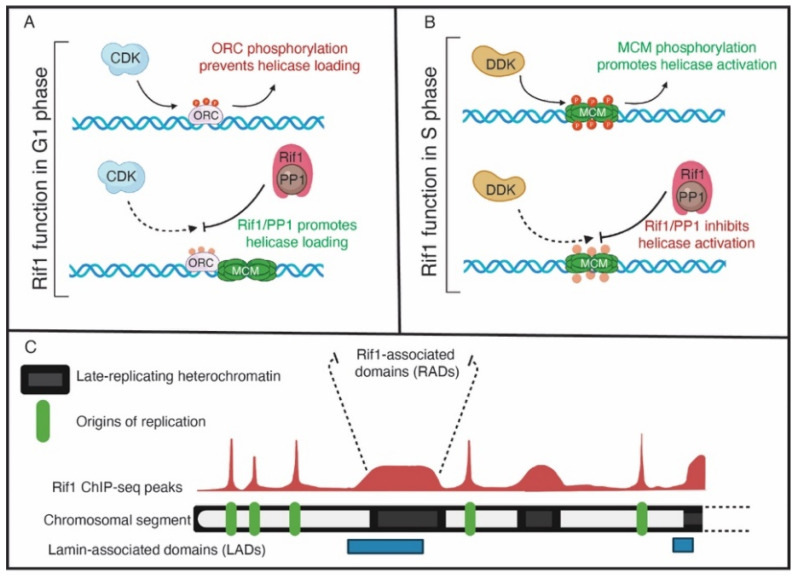
The functions of Rif1. (**A**) In G1 phase, Rif1 may reverse the CDK-mediated phosphorylation of ORC to promote ORC-dependent loading of MCMs. (**B**) In S phase, Rif1 opposes DDK-mediated MCM phosphorylation to inhibit helicase activation and origin activation in late-replicating genomic regions. (**C**) A representative view of Rif1 genomic binding on a chromosomal arm. Green boxes denote origins of replication, where Rif1 genomic binding has a sharp, well-defined peaks. Black boxes indicate regions of heterochromatin, where Rif1 binds to broad domains with a lower signal intensity compared to origins. The broad Rif1 binding domains also have overlap with lamin-associated domains, indicated by blue boxes.

In addition to Sld3, Cdc45 and Dpb11, Rif1 appears to regulate ORC1 activity [55]. An unbiased phosphoproteomic screen revealed that, in addition to MCMs, ORC1 is hyper phosphorylated upon Rif1 depletion in human cells [55]. Additionally, the level of chromatin-bound ORC1 is reduced upon Rif1 depletion. The consequence of this is a reduction in MCM loading in G1 phase of the cell cycle. This work revealed that Rif1 protects ORC1 from phosphorylation as phosphorylation of ORC1 targets it for degradation [55]. Ironically, Rif1 appears to target ORC1 in G1 phase to promote helicase loading and targets MCMs in S phase to prevent helicase activation [55] (Figure 2A,B). While this may initially seem counterintuitive, it appears that Rif1 functions in two phases of the cell cycle to ensure enough helicases are loaded while preventing excessive helicase activation. In this regard, Rif1 is a major regulator of the overall DNA replication program.

## 4. Rif1 Activity Is Regulated during Development

In spite of different species-specific functions and mechanisms, Rif1 plays an important role in governing the global RT program from yeast to mammals [22,32,33,34,36,63]. The effect Rif1 has on global RT, however, varies among species and depends on developmental state. In budding yeast and fission yeast, Rif1 affects RT of 65% and 30% of the origins, respectively [32,63]. In Drosophila, 8–30% of genome-wide RT depends on Rif1 depending on cell type [36,64]. In mammalian cells, Rif1-mediated control of RT can range from ~23% to ~100% depending on cell type with human embryonic stem cells (hESCs) showing the most significant dependence on Rif1 for RT [22,33,65]. In both Drosophila and human cells, RT is also sensitive to the dosage of Rif1 [22,36]. While it seems logical to assume that, upon loss of Rif1 function, there is a predominate transition from late to early (LtoE) replication throughout the genome, often the fraction of the genome that transitions from EtoL and LtoE are equal [22,32,36,63,65]. It is still unclear why such a large fraction of the genome transition from EtoL based on the Rif1’s ability to prevent helicase activation. However, EtoL changes could be driven through indirect effects of LtoE RT changes within such a large fraction of the genome [36]. Determining the specific mechanism driving the Rif1-dependent changes in replication timing, notably the EtoL transitions, still remains a gap in knowledge to be addressed by the field. 

Understanding how Rif1 activity is regulated during development and differentiation could reveal the molecular basis for how cell-type-specific RT programs are established. One powerful system to directly interrogate how Rif1 activity is modulated during development is the early Drosophila embryo. The first 14 cell cycles in the Drosophila embryo are rapid and tightly orchestrated [66]. S phase in the first nine cell cycles is 3–4 min in length [67]. Starting in cycle 9, S phase gradually slows until cell cycle 14 where S phase more dramatically slows to 50 min [68]. The slowing of S phase in cycle 14 is driven by the onset of RT and a pattern of late replication where heterochromatin is exclusively replicated [68]. This provides a unique opportunity to study the factors and processes that drive the onset of RT. Critically, one factor that significantly contributes to the slowing of S phase in cycle 14 and the onset of late replication is Rif1 [35]. In fact, S phase is ~60% faster in cell cycle 14 in Rif1 mutant embryos and the characteristic pattern of late replication is lost [35]. Importantly, Rif1 localization patterns anticipate the establishment of RT in cycle 14. Rif1 localizes to satellite sequences in cycle 14, but dissociates prior to their replication [35]. What makes this such a powerful system is that Rif1 is present in cycles 1–13 but is held inactive. Rif1 appears to be activated prior to cycle 14 [35]. What could drive this switch in Rif1 activity? Rif1 contains CDK consensus sites and is heavily phosphorylated in the early embryo, raising the possibility that high levels of CDK activity in the early embryo keep Rif1 inactive [35]. Consistent with this hypothesis, expression of a Rif1 protein with all CDK sites mutated to alanine (phosphomutant) blocks replication resulting in mitotic errors [35]. This suggests that phosphorylation acts as a molecular switch to control Rif1 activity. Cells are sensitive to the level of Rif1 expression and overexpression of wild-type Rif1 is detrimental to cells, which could be a caveat to this experiment [35]. In human cells, Rif1-phosphorylation is also dependent on CDK1 [69]. In Xenopus egg extract, which mimics the first S phase after fertilization, depletion of Rif1 or Rif1-CTD (C-terminal domain that contains PP1-binding site) results in less PP1 on chromatin and a reduced rate of MCM-phosphorylation [69,70]. This suggests that Rif1 has the potential to regulate replication in the earliest stages of vertebrate development. Interestingly, in Xenopus, Polo-like kinase 1 (Plk1) can phosphorylate Rif1 near its PP1-binding site, suggesting that Rif1 is the target of multiple cell-cycle-regulated kinases [70].

Work in Drosophila shows that Rif1 functions in S phase beyond the control of helicase activation. In polyploid cells, specific regions of the genome are repressed for DNA replication in a process known as underreplication, resulting in reduced copy number relative to overall ploidy [71,72]. Interestingly, in both Drosophila and mammals, Rif1 is a key regulator of underreplication [64,73,74]. In Drosophila, the Suppressor of UnderReplication (SuUR) protein is recruited to replication forks and inhibits their progression, resulting in underreplication [75]. Surprisingly, SuUR associates with Rif1 and can recruit Rif1 to replication forks [73]. Given that underreplication is completely dependent on Rif1, this suggests that Rif1 can function at a replication fork to inhibit fork progression [73]. How can Rif1 inhibit an active replication fork? Perhaps Rif1/PP1 could de-phosphorylate MCMs as part of the active helicase, resulting in its destabilization [59]. This work highlights Rif1’s potentially diverse functions and raises the possibility that Rif1/PP1 activity could be used for a variety of other nuclear-related processes. 

## 5. Rif1 Dynamically Associates with Chromatin through the Cell Cycle and Development

Understanding how and where Rif1 is localized to chromatin is an important step in understanding Rif1 function. Immunofluorescent (IF) and live cell imaging studies reveal that, in metazoans, Rif1 dynamically associates with chromatin during S phase [33,34,35,37]. The exact nature of Rif1 localization with respect to DNA replication is not as clear. In mouse and human cells, Rif1 localizes to chromocenters or DNaseI insoluble chromatin and during mid S phase Rif1 colocalizes with BrdU, a marker of DNA replication [34,37]. Other studies in mouse cells and Drosophila embryos, however, show that Rif1 disassociates from chromatin prior to the formation of replication foci [33,35]. Given that loss of Rif1 activity often results in LtoE RT switches within heterochromatin, it is tempting to speculate that Rif1 is somehow recruited to heterochromatin where it prevents helicase activation and thus promotes late replication of heterochromatin. While this is likely true, it also over simplifies Rif1 function throughout the genome. For example, genome-wide measures of Rif1-dependent RT reveal changes throughout the genome and not just within heterochromatin. In addition, loss of Rif1 function causes EtoL RT switches, not just LtoE. It is possible that, in addition to heterochromatin, Rif1 localizes sites throughout the euchromatic portion of the genome. These sites, however, may be hard to visualize by IF given the strong signal at heterochromatin. 

High-resolution localization of Rif1 has been measured using both ChIP-seq and CUT&RUN [22,32,55,65,76,77]. Features of Rif1 genomic binding, however, vary depending on the organism. Early work in budding yeast revealed that Rif1 binds to chromatin primarily at telomeres and is dependent on Rif1’s binding partner: Rap1 [30]. Beyond budding yeast, there are seem to be two characteristics of Rif1 binding: Rif1 binds to late-replicating genomic regions [22,65] and Rif1 binding is enriched at origins of replication [32]. 

In fission yeast, Rif1 binds telomere proximal regions and late-replicating origins of replication within subtelomeric regions [32]. In G1 and early S phase cells, Rif1 also binds to both early and late-replicating origins of replication throughout the genome. Interestingly, in fission yeast, Rif1 binds to centromeres during M phase and remains bound until the completion of S phase [32]. Further work in fission yeast also revealed that Rif1 binding sites are enriched for a consensus sequence. This Rif1 consensus sequence contains G-quadruplex-like structures, and G-quadruplexes are necessary for Rif1 binding [77].

Consistent with Rif1’s telomere-specific functions in budding yeast, Rif1 also shows strong binding to telomeres [78]. Rif1’s association to telomeres is dependent on Rap1, which targets Rif1 to yeast telomeres [30,76]. In budding yeast, however, Rif1 also binds to genomic regions independently of Rap1. Specifically, Rif1 associates with many replication origins both near and distant to telomeres [76], similar to fission yeast [32]. Surprisingly, Rif1 also associates with the coding regions of highly transcribed genes independently of Rap1, and the biological reason for this observation still remains unknown [76]. 

In mouse embryonic stem cells, Rif1’s genomic distribution overlaps primarily with late-replicating regions and is depleted from early replicating regions, which is consistent with the hypothesis that Rif1 is recruited to chromatin to prevent helicase activation [55,58,59]. In addition, Rif1 binds large genomic domains termed Rif1-associated domains or RADs. RADs show significant overlap with Lamin associated domains (LADs), which are associated with the nuclear lamina and tend to be late-replicating [65]. Besides the broad RADs, a smaller fraction of Rif1 forms more distinct peaks. Only a subset of these peaks, however, are associated with potential replication origins [65]. These sites are often in early replicating regions that are associated with transcription start sites and have high GC content with the possibility of forming G4 quadruplexes. Critically, while Rif1 appears to bind and regulate individual replication origins in fission yeast [32], Rif1 appears to act more broadly at the domain level to regulate replication in mammals [65].

More recently, Rif1 binding has been profiled using CUT&RUN in human embryonic cell lines. Similar to mouse cells [65], Rif1 was enriched within late-replicating genomic regions and bound broad domains [22]. Importantly, Rif1 binding occurred at genomic regions that became de-regulated in their replication timing upon loss of Rif1 function [22]. Why Rif1 forms broad domains within late-replicating regions across species while also binding specifically to replication origins is not understood. Perhaps there are multiple populations of Rif1: Rif1 targeted to chromatin domains to promote late replication by opposing helicase activation and Rif1 targeted to replication origins and transcription start sites to perform an alternative regulatory function. In this way, Rif1 could perform different functions depending on chromatin context. The underlying factors necessary for Rif1 recruitment to chromatin are still unclear. 

## 6. Rif1 and Nuclear Organization

RT is highly correlated with the spatial organization of the genome. It has long been known that regions of the genome which are early replicating tend to localize towards the center of the nucleus, while the late-replicating regions are often localized to the nuclear periphery [79,80]. This relationship is not absolute, however, since altering nuclear position does not always cause predictable changes in RT [81]. Based on chromatin capture techniques, the genome can be classified into two compartments—A, enriched with open chromatin and gene-rich loci, and B, comprised of more densely packed gene-poor regions [82]. RT correlates with A/B compartment structure similar as expected based on cytological studies; early-replicating regions line up with the A compartment and late-replicating regions correlated with the B compartment [83]. 

RT is also highly correlated with nuclear architecture. Hi-C-based chromosome capture experiments have classified megabase-scale self-associating genomic folding that can range from as the primary structural units of chromatin domains [82,84,85]. These spatially organized domains are commonly called Topologically Associated Domains (TADs). TADs serve as the units for the genomic-level organization of the chromosomes that remain stable through cell divisions and diverse cell types. A TAD can be defined as a self-interacting genomic region, with two basic features of organization—self-association and neighbor-insulation. A study of 18 human and 13 mouse cell types mapped the genomic boundaries of TADs and Replication Domains (RD; contiguous genomic regions with similar RT status) by Hi-C and discovered that they share a near 1:1 correlation [86].

**Figure 3:**
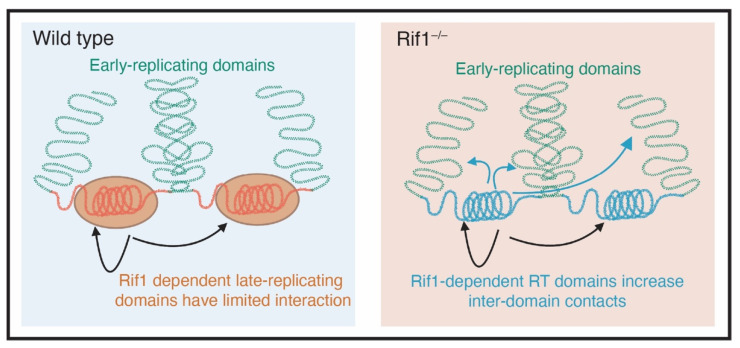
Rif1 mediates nuclear organization. In cells that are wild type for *Rif1* (blue box), early and late replicating domains are separated from each other (green and red DNA respectively). The late replicating domains, which are coated with Rif1, have limited physical interactions. In cells that are mutant for *Rif1*, there is an increase in physical interactions between genomic domains that depend on Rif1 to maintain their replication timing (Adapted from Foti et al., 2016 [65]).

While it is clear that RT is highly correlated by nuclear organization and structure, it is less clear what underlying molecular mechanisms drive these correlations. Recent work, however, has suggested that Rif1 may provide a link between nuclear organization and RT. First, Rif1 associates with Lamin, thus providing a link between Rif1-associated domains (RADs) and the nuclear periphery [33,65]. Second, Rif1 has a critical role in promoting 3D nuclear organization [65,87] (Figure 3). If Rif1 is a key link between RT and nuclear organization, then a powerful tool to understand the connection between Rif1-dependent RT and nuclear organization would be a separation of function mutant. The Rif1^PP1^ mutant (with a mutated PP1-binding motif) would be a good candidate to separate Rif1 functions. Unfortunately, however, the Rif1^PP1^ mutant disrupts both RT and nuclear organization almost identically to a Rif1 null mutant in mouse embryonic stem cells (ESCs) where these experiments have been performed [87]. In mouse ESCs, nuclear organization and RT are differentially sensitive to Rif1 dosage [87]. Cells hemizygous for Rif1 have normal RT timing but altered nuclear organization, suggesting that nuclear organization, but not RT, is sensitive to Rif1 dosage [87]. It is surprising, however, that RT is not sensitive to Rif1 dosage in mouse embryonic stem cells while RT is sensitive to Rif1 dosage in both Drosophila and human embryonic stem cells [22,36]. Regardless, one interpretation of this work is that Rif1-dependent nuclear organization is independent from Rif1’s role in regulating RT. This would be consistent with 4C data in mouse cells showing that depletion of Rif1 in G1 (prior to execution of an altered RT program) causes an increase in inter-TAD interactions in G1, further arguing that Rif1 has a direct role on controlling nuclear organization independent of RT [65]. A similar developmental timeline of emergence [35,88] and a high degree of overlap between boundaries of TADs and RDs [86] hints at a coordinated function. With the emerging role of RT in the maintenance of epigenetic landscape [22], however, any experimental data in support of RT and nuclear architecture being synchronized to govern gene expression remain to be seen.

## 7. Future Directions and Outstanding Questions

Rif1 has many functions, from controlling helicase activation to regulation of nuclear architecture, ORC1 activity, and replication fork progression [55,65,73]. Altogether, the primary proposed mechanism for Rif1 is to oppose the DDK-mediated phosphorylation of loaded helicases to prevent helicase activation within specific regions of the genome. More recently, Rif1, and subsequently RT, have been linked to the maintenance of epigenetic state. Rif1 also has a number of additional functions in DNA repair and telomere length control that we did not discuss [30,39,43,44,45,46,49,89]. All of these functions are still primed for further investigation to ultimately understand how Rif1 function is controlled throughout the cell cycle, tissue differentiation, and organismal development to ensure accurate and timely duplication of the genome. Beyond functions, the control of Rif1 activity through phosphorylation should also be a fruitful area of research. Further work is needed to explore how Rif1 activity is during development and across species. While we have discussed specific aspects of Rif1-dependent control of RT, many questions still remain. For example, how does Rif1/PP1 selectively target helicases within specific genomic regions? What other replication factors does Rif1/PP1 directly target? What molecular mechanism does Rif1 use to control nuclear organization? What is the exact relationship between Rif1-dependent nuclear organization and RT? How is Rif1 activity controlled during development to establish cell-type-specific RT programs? Answering these, and other key questions, will generate a better understanding of how RT contributes to diverse processes from genome maintenance to organismal development. 

## Data Availability

Not applicable.
